# Inhibition of dengue virus replication by novel inhibitors of RNA-dependent RNA polymerase and protease activities

**DOI:** 10.1080/14756366.2017.1355791

**Published:** 2017-08-04

**Authors:** Sveva Pelliccia, Yu-Hsuan Wu, Antonio Coluccia, Giuseppe La Regina, Chin-Kai Tseng, Valeria Famiglini, Domiziana Masci, John Hiscott, Jin-Ching Lee, Romano Silvestri

**Affiliations:** a Department of Drug Chemistry and Technologies, Sapienza University of Rome, Laboratory affiliated to Istituto Pasteur Italia – Fondazione Cenci Bolognetti, Roma, Italy;; b Institute of Basic Medical Sciences, College of Medicine, National Cheng Kung University, Tainan, Taiwan;; c Center of Infectious Disease and Signaling Research, College of Medicine, National Cheng Kung University, Tainan, Taiwan;; d Istituto Pasteur Italia – Fondazione Cenci Bolognetti, Roma, Italy;; e Department of Biotechnology, College of Life Science, Kaohsiung Medical University, Kaohsiung, Taiwan;; f Graduate Institute of Natural Products, College of Pharmacy, Kaohsiung Medical University, Kaohsiung, Taiwan;; g Research Center for Natural Products and Drug Development, Kaohsiung Medical University, Kaohsiung, Taiwan;; h Graduate Institute of Medicine, College of Medicine, Kaohsiung Medical University, Kaohsiung, Taiwan

**Keywords:** DENV inhibitors, RdRp, NS3 protease, ICR-suckling mouse, synergy

## Abstract

Dengue virus (DENV) is the leading mosquito-transmitted viral infection in the world. With more than 390 million new infections annually, and up to 1 million clinical cases with severe disease manifestations, there continues to be a need to develop new antiviral agents against dengue infection. In addition, there is no approved anti-DENV agents for treating DENV-infected patients. In the present study, we identified new compounds with anti-DENV replication activity by targeting viral replication enzymes – NS5, RNA-dependent RNA polymerase (RdRp) and NS3 protease, using cell-based reporter assay. Subsequently, we performed an enzyme-based assay to clarify the action of these compounds against DENV RdRp or NS3 protease activity. Moreover, these compounds exhibited anti-DENV activity *in vivo* in the ICR-suckling DENV-infected mouse model. Combination drug treatment exhibited a synergistic inhibition of DENV replication. These results describe novel prototypical small anti-DENV molecules for further development through compound modification and provide potential antivirals for treating DENV infection and DENV-related diseases.

## Introduction

Dengue virus (DENV) is responsible of worldwide arthropodborne viral infection, which globally represents a serious human health concern. DENV is the etiological agent of disease in more than hundred countries with up to 3 billion people exposed to the risk of infection in the tropical regions^1–3^. DENV has expanded its global range with sustained outbreaks in South America and Asia, with these epidemics accompanied by increased disease severity.

DENV are single-stranded, positive sense RNA viruses belonging to the *Flaviviridae* family. The DENV family can be viewed as falling in four related, but antigenically distinct, DENV 1–4 serotypes. The carriers of DENV to humans are the mosquitoes *Aedes aegypti* and *Aedes albopictus.* The DENV infection causes a variety of illness, including asymptomatic or subclinical disease, dengue fever (DF) symptoms and the most severe dengue haemorrhagic fever (DHF) and dengue shock syndrome (DSS) which cause millions of infections around the world^4–8^. DENV has a 10.7 kb, positive-sense RNA genome with 5′- and 3′-untranslated regions flanking a polyprotein that encodes three structural (C, prM/M and E) and seven non-structural (NS1, NS2A, NS2B, NS3, NS4A, NS4B and NS5) proteins. prM and E structural proteins are the primary antigenic targets of the humoural immune response in humans^9–12^. DENV NS3 is a multifunctional protein, which contains protease, helicase and triphosphatase domain. The *N*-terminal amino acids (residue 1–184) of NS3 is responsible for protease activity. NS2B serves as a cofactor of NS3 protease and forms complex with NS3; the central amino acid hydrophilic domain (residue 49–92) of NS2B is critical for cofactor activity^13^. DENV replication also requires the non-structural protein 5 (NS5) which is the essential RNA-dependent RNA polymerase (RdRp) activity^14^. DENV NS5 RdRp has proven to be a promising target for direct-acting antiviral (DAA) drug development, because it is structurally conserved among the four DENV serotypes, and NS5 RdRp has no enzymatic counterpart in mammalian cells^15^.

Currently, no licensed antiviral drugs are available to block DENV infection, and while vector control efforts remain the only means to stop the spread of the infection, they have not successfully inhibited annual epidemic outbreaks throughout the tropics^16^. Recently, a live-attenuated DENV vaccine^17^ based on the yellow fever virus 17 D backbone was licensed for use in the Philippines, Brazil and Mexico. However, the serotype-specific efficacy of the DENV vaccine is varying, and the long-term protection and safety of this vaccine still need more investigation^18^. In the present study, we have focused on the identification of potential anti-DENV inhibitors by targeting the enzymatic activities of the NS5 RdRp polymerase and NS3 protease *in vitro* and *in vivo*^19–21^. As part of a continuation of our studies^22–24^, we developed new pyrazole derivatives^25^ as potential DENV NS5 RdRp inhibitors (Table 1). In addition, we carried out virtual screening (VS) studies on the NS2B/NS3 protease to design and synthesise new DENV NS3 protease inhibitors (Table 2). In sum, we identified five compounds that exhibited anti-DENV replication activity without cytotoxicity; two compounds exhibited anti-DENV activity in DENV-infected ICR-suckling mouse model. Interestingly, combination treatment with compounds, respectively, targeting NS5 RdRp polymerase and NS3 protease, demonstrated a synergistic inhibitory effect on DENV replication.

**Table 1.** Activity of Compounds **1–3** against DENV-2 Replication and DENV-2 NS5 RdRp.

		DENV-2^a^				
		RNA			NS5 RdRp	
	Structure	CC_50_^b^(μM)	EC_50_^c^ ± SD (μM)	SI^d^	Cell-based EC_50_^e^ ± SD (μM)	Enzyme-based EC_50_^f^ ± SD (μM)
**1**		196	11.7 ± 0.2	16.7	8.1 ± 0.3	7.8 ± 0.3
**2**		>200	7.6 ± 0.4	>26.3	7.2 ± 0.4	5.3 ± 0.2
**3**		>200	5.7 ± 0.3	>35.1	6.0 ± 0.3	4.9 ± 0.2

a Data are mean values of two to three independent experiments each one in triplicate.

b CC_50_: half maximal cytotoxicity concentration.

c EC_50_ (DENV-2 RNA): half maximal effective concentration. Huh-7 cells were infected with DENV-2 and followed by RdRp inhibitors treatment for 3 days. The cell lysates were collected to analyse DENV RNA synthesis by qRT-PCR with specific primers targeting NS5.

d SI: selectivity index calculated as CC_50_/EC_50_ ratio.

e EC_50_ (cell-based DENV-2 NS5 RdRp): cell-based RdRp reporter assay. The Huh-7 cells were transiently expressed p(+)RLuc-(–)DV-UTRΔC-FLuc and DENV NS5 expression vector pcDNA-NS5-Myc.

f EC_50_ (enzyme-based DENV-2 NS5 RdRp): enzyme-based RdRp activity assay. The (−) 3′UTR RNA was incubated with RdRp polymerase protein and CTP, GTP, UTP and BBT-ATP. The fluorescence signal was measured at excitation wavelength of 422 nm and emission wavelength of 566 nm, respectively.

**Table 2.** Activity of DENV inhibitors **4** and **5** against DENV-2 replication.

		DENV-2^a^				
		RNA			NS3	
	Structure	CC_50_^b^ (μM)	EC_50_^c^ ± SD (μM)	SI^d^	Cell-based EC_50_^e^ ± SD (μM)	Enzyme-based EC_50_^f^ ± SD (μM)
**4**		181	4.6 ± 0.3	39.3	6.7 ± 0.2	4.7 ± 0.3
**5**		>200	7.3 ± 0.3	27.4	7.9 ± 0.6	6.9 ± 0.4

a Data are mean values of two to three independent experiments each one in triplicate.

b CC_50_: half maximal cytotoxicity concentration.

c EC_50_ (DENV-2 RNA): half maximal effective concentration. Huh-7 cells were infected with DENV-2 and followed by RdRp inhibitors treatment for 3 days. The cell lysates were collected to analyse DENV RNA synthesis by qRT-PCR with specific primers targeting NS5.

d SI: selectivity index calculated as CC_50_/EC_50_ ratio.

e EC_50_ (cell-based DENV-2 NS3 protease): Huh-7 cells were transfected with pEG(MITA)SEAP and pcDNA-NS2B-GSG-NS3-Myc followed by incubation of each compound. The SEAP activity was measured by Phodpha-Light assay kit after 3 days post incubation.

f EC_50_ (enzyme-based DENV-2 NS3 protease). The 7-amino-4-methylcoumarin (AMC) fluorophore-linked peptide substrate Boc-GRR-AMC (Bachem, USA) was incubated with DENV NS2B/NS3pro protein and compound. The fluorescence signal was detected at excitation wavelength of 380 nm and emission wavelength of 465 nm, respectively.

## Materials and methods

### Chemistry

Microwave (MW)-assisted reactions were performed on a CEM Discover SP single-mode reactor equipped with Explorer 72 autosampler, controlling the instrument settings by PC-running CEM Synergy 1.60 software (Matthews, NY, USA). Closed vessel experiments were carried out in capped MW-dedicated vials (10 ml) with cylindrical stirring bar (length 8 mm, diameter 3 mm). Stirring, temperature, irradiation power, maximum pressure (*P*_max_), PowerMAX (simultaneous cooling while heating), ActiVent (simultaneous venting while heating), and ramp and hold times were set as indicated. Temperature of the reaction was monitored by an external fibre optic temperature sensor. After completion of the reaction, the mixture was cooled to 25 °C via air jet cooling. Organic solutions were dried over anhydrous sodium sulphate. Evaporation of the solvents was carried out on a Bu¨chi Rotavapor R-210 equipped with a Bu¨chi V-850 vacuum controller and a Bu¨chi V-700 vacuum pump. Column chromatography was performed on columns packed with silica gel from Macherey-Nagel (70 − 230 mesh). Silica gel thin-layer chromatography (TLC) cards from Macherey-Nagel (silica gel pre-coated aluminium cards with fluorescent indicator visualisable at 254 nm) were used for TLC. Developed plates were visualised with a Spectroline ENF 260 C/FE UV apparatus. Melting points (mp) were determined on a Stuart Scientific SMP1 apparatus and are uncorrected. Infrared spectra (IR) were run on a PerkinElmer Spectrum 100 FT-IR spectrophotometer equipped with universal attenuated total reflectance (ATR) accessory and IR data acquired and processed by PerkinElmer Spectrum 10.03.00.0069 software. Band position and absorption ranges are given in cm^−1^. Proton nuclear magnetic resonance (^1^H NMR) spectra were recorded a Bruker Avance (400 MHz) spectrometer in the indicated solvent and corresponding fid files were processed by MestreLab Research SL MestreReNova 6.2.1–769 software (Santiago de Compostela, Spain). Chemical shifts are expressed in *δ* units (ppm) from tetramethylsilane. Elemental analyses of biologically evaluated compounds were found to be within ±0.4% of the theoretical values and their purity was found to be >95% by high-pressure liquid chromatography (HPLC). The HPLC system used Thermo Fisher Scientific Dionex UltiMate 3000, consisted of a SR-3000 solvent rack, a LPG-3400SD quaternary analytical pump, a TCC-3000SD column compartment, a DAD-3000 diode array detector and an analytical manual injection valve with a 20 µL loop. Samples were dissolved in acetonitrile (1 mg/mL). HPLC analysis was performed by using a Thermo Fisher Scientific Acclaim 120 C18 column (5 µm, 4.6 mm × 250 mm) at 25 ± 1 °C with an appropriate solvent gradient (acetonitrile/water), flow rate of 1.0 mL/min and signal detector at 206, 230, 254 and 365 nm. Chromatographic data were acquired and processed by Thermo Fisher Scientific Chromeleon 6.80 SR15 Build 4656 software (Waltham, MA, USA). 

*General procedure for preparation of compounds****1, 3****and****8****. Ethyl 5–(4-chloro-N-((4-chlorophenyl)sulphonyl)phenylsulphonamido)-1-methyl-1H-pyrazole-4-carboxylate (****1****).* 4-Chlorobenzenesulphonyl chloride (0.15 g, 0.72 mmol) was added to solution of commercially available **6** (0.10 g, 0.6 mmol) in pyridine (2 mL) while stirring. The reaction was stirred at 60 °C overnight. The mixture was neutralised with 1 N HCl and extracted with ethyl acetate. The organic layer was washed with brine, dried and filtered. Removal of the solvent gave a residue that was purified by column chromatography (silica gel, *n*-hexane:ethyl acetate = 2:1 as eluent) to furnish **1** (0.10 g, 33%), mp 132–135 °C (from ethanol). ^1^H NMR (DMSO-d_6_): *δ* 0.96 (*t*, *J* = 6.2 Hz, 3H), 3.52 (*s*, 3H), 3.71–3.77 (*m*, 2H), 7.81 (d, *J* = 8.0 Hz, 4H), 7.94 (d, *J* = 7.9 Hz, 4H), 8.06 ppm (*s*, 1H). FT-IR (ATR): ν 1176, 1391, 1709 cm^−1^. Anal. calcd. for C_19_H_17_Cl_2_N_3_O_6_S_2_ (518.38): C, 44.02; H, 3.31; N, 8.11; Cl, 13.68; S, 12.37. Found: C, 43.78; H, 3.26; N, 7.84; Cl, 13.36; S, 12.02.

*Ethyl 5–(4-nitro-N-((4-nitrophenyl)sulphonyl)phenylsulphonamido)-1-phenyl-1H-pyrazole-4-carboxylate (****3****).* Was synthesised as **1** starting from **7**^26^ and 4-nitrobenzensulphonyl chloride. Yield 79%, mp 230–232 °C (from ethanol). ^1^H NMR (DMSO-d_6_): *δ* 1.01 (*t*, *J* = 7.0, 3H), 3.70–3.76 (*m*, 2H), 7.32–7.38 (*m*, 3H), 7.48 (d, *J* = 6.9 Hz, 2H), 8.04 (d, *J* = 8.9 Hz, 4H), 8.33–8.36 ppm (*m*, 5H). FT-IR (ATR): ν 1167, 1336, 1597, 1706 cm^−1^. Anal. calcd. for C_24_H_19_N_5_O_10_S_2_ (601.06): C, 47.92; H, 3.18; N, 11.64; S, 10.66. Found: C, 47.78; H, 3.11; N, 11.39; S, 10.42.

*Ethyl 1-methyl-5–(4-nitro-N-((4-nitrophenyl)sulphonyl)phenylsulphonamido)-1H-pyrazole-4-carboxylate (****8****).* Was synthesised as **1** starting from **6** and 4-nitrobenzenesulphonyl chloride. Yield 33%, mp 220–222 °C (from ethanol). ^1^H NMR (DMSO-d_6_): *δ* 0.93 (*t*, *J* = 7.1 Hz, 3H), 3.54 (*s*, 3H), 3.69–3.74 (*m*, 2H), 8.11 (*s*, 1H), 8.23 (d, *J* = 8.9 Hz, 4H), 8.52 ppm (d, *J* = 8.9 Hz, 4H). FT-IR (ATR): ν 1173, 1349, 1528, 1717 cm^−1^. Anal. calcd. for C_19_H_17_N_5_O_10_S_2_ (539.49): C, 42.30; H, 3.18; N, 12.98; S, 11.89. Found: C, 42.12; H, 3.14; N, 12.60; S, 11.70.

*Ethyl 1-methyl-5–(4-nitrophenylsulphonamido)-1H-pyrazole-4-carboxylate (****9****).* A mixture of **8** (0.032 g, 0.06 mmol) and lithium hydroxide monohydrate (0.01 g, 0.24 mmol) in THF/H_2_O (1:1, 5 mL) was heated at 50 °C for 15 min, cooled, made acid with 1 N HCl aqueous solution (pH ≈ 5) and extracted with ethyl acetate. The organic layer was washed with brine, dried and filtered. Removal of the solvent gave **9** (0.02 g, 95%), mp 150–152 °C (from ethanol). ^1^H NMR (DMSO-d_6_): δ 0.98 (*t*, *J* = 7.08, 3H), 3.71–3.76 (*m*, 5H), 7.78 (*s*, 1H), 7.87 (d, *J* = 8.9 Hz, 2H), 8.40 (d, *J* = 8.9 Hz, 2H), 11.01 ppm (br s, disappeared on treatment with D_2_O, 1H). FT-IR (ATR): ν 1170, 1347, 1532, 1709 cm^−1^. Anal. calcd. for C_13_H_14_N_4_O_6_S (354.34): C, 44.07; H, 3.98; N, 15.81; S, 9.05. Found: C, 43.84; H, 3.92; N, 15.62; S, 8.91.

*Ethyl 5–(4-aminophenylsulphonamido)-1-methyl-1H-pyrazole-4-carboxylate (****10****).* A mixture of **9** (0.14 g, 0.4 mmol) and tin(II) chloride dihydrate (0.27 g, 1.2 mmol) in ethyl acetate (15 ml) was heated at reflux for 3 h. After cooling, the mixture was made basic (pH ≈ 8) with a saturated aqueous solution of NaHCO_3_ and filtered. The layers were separated and the organic one was washed with brine, dried and filtered. Removal of the solvent gave a residue that was purified by column chromatography (silica gel, dichloromethane:ethanol = 9.8:0.2 as eluent) to furnish **10** (0.06 g, 50%), mp 118–120 °C (from ethanol). ^1^H NMR (DMSO-d_6_): δ 1.09 (*t*, *J* = 7.1 Hz, 3H), 3.66 (*s*, 3H), 3.83–3.88 (*m*, 2H), 6.02 (*s*, 2H), 6.52 (d, *J* = 8.7 Hz, 2H), 7.20 (d, *J* = 8.7 Hz, 2H), 7.72 (*s*, 1H), 9.71 ppm (br s, disappeared on treatment with D_2_O, 1H). FT-IR (ATR): ν 1152, 1378, 1698, 3397 cm^−1^.

*Ethyl 5–(4-(1H-pyrrol-1-yl)phenylsulphonamido)-1-methyl-1H-pyrazole-4-carboxylate (****11****).* A mixture of **10** (0.05 g, 0.15 mmol) and 2,5-dimethoxytetrahydrofuran (0.02 g, 0.02 mL, 0.15 mmol) in glacial acetic acid (1 mL) was heated at reflux for 30 min. The solvent was removed *in vacuo*, and after cooling water and ethyl acetate were added. Layers were separated and the organic one was washed with a saturated aqueous solution of NaHCO_3_, brine and dried. Removal of the solvent gave a residue that was purified by column chromatography (silica gel, *n*-hexane:ethyl acetate = 1:1 as eluent) to furnish **11** (0.035 g, 66%), mp 200–203 °C (from ethanol). ^1^H NMR (DMSO-d_6_): *δ* 0.97 (*t*, *J* = 7.1 Hz, 3H), 3.73–3.77 (*m*, 5H), 6.32 (*t*, *J* = 2.2 Hz, 2H), 7.49 (*t*, *J* = 2.2 Hz, 2H), 7.62 (d, *J* = 8.8 Hz, 2H), 7.76–7.78 (*m*, 3H), 10.48 ppm (br s, disappeared on treatment with D_2_O, 1H). FT-IR (ATR): ν 1132, 1336, 1702, 3139 cm^−1^. Anal. calcd. for C_17_H_18_N_4_O_4_S (374.41): C, 54.53; H, 4.85; N, 14.96; S, 8.56. Found: C, 54.28; H, 4.79; N, 14.69; S, 8.22.

*Ethyl 5–(4-(2-benzoyl-1H-pyrrol-1-yl)phenylsulphonamido)-1-methyl-1H-pyrazole-4-carboxylate (****2****).* A mixture of **11** (0.06 g, 0.14 mmol), benzoyl chloride (0.02 g, 0.016 mL, 0.14 mmol) and anhydrous aluminium chloride (0.02 g; 0.14 mol) in anhydrous 1,2-dichloroethane (3 mL) was placed into the MW cavity (closed vessel mode, Pmax = 250 psi). A starting MW irradiation of 70 W was used, the temperature being ramped from 25 to 110 °C, while stirring. Once 110 °C was reached, taking about 1 min, the reaction mixture was held at this temperature for 4 min. The reaction mixture was quenched on 1 N HCl aqueous solution/crushed ice and extracted with dichloromethane. The organic layer was washed with brine, dried and filtered. Removal of the solvent gave a residue that was purified by column chromatography (silica gel, *n*-hexane:acetone = 2:1 as eluent) to furnish **2** (0.04 g, 71%), mp 136–140 °C (from ethanol). ^1^H NMR (DMSO-d_6_): *δ* 1.07 (*t*, *J* = 7.1 Hz, 3H), 3.67 (*s*, 3H), 3.87–3.93 (*m*, 2H), 6.43–6.45 (*m*, 1H), 6.87–6.89 (*m*, 1H), 7.45–7.46 (*m*, 1H), 7.52–7.56 (*m*, 4H), 7.63–7.69 (*m*, 3H), 7.78 (*s*, 1H), 7.82–7.84 (*m*, 2H) 10.60 ppm (br s, disappeared on treatment with D_2_O, 1H). FT-IR (ATR): ν 1166, 1347, 1595, 1713, 3102 cm^−1^. Anal. calcd. for C_24_H_22_N_4_O_5_S (478.52): C, 60.24; H, 4.63; N, 11.71; S, 6.70. Found: C, 59.92; H, 4.58; N, 11.46; S, 6.46. Further elution with the same eluent gave *ethyl 5–(4-(3-benzoyl-1H-pyrrol-1-yl)phenylsulphonamido)-1-methyl-1H-pyrazole-4-carboxylate* (0.02 g, 28%), mp 216–218 °C (from ethanol). ^1^H NMR (DMSO-d_6_): *δ* 0.97 (*t*, *J* = 7.0 Hz, 3H), 3.73–3.79 (*m*, 5H), 6.80–6.82 (*m*, 1H), 7.53–7.57 (*m*, 2H), 7.62–7.68 (*m*, 4H), 7.76 (*s*, 1H), 7.84–7.86 (d, *J* = 8.4 Hz, 2H), 7.93–7.96 (d, *J* = 9.0 Hz, 2H), 8.04 (*s*, 1H), 10.58 ppm (br s, disappeared on treatment with D_2_O, 1H). FT-IR (ATR): ν 1166, 1280, 1642, 1706, 3132 cm^−1^. Anal. calcd. C_24_H_22_N_4_O_5_S (478.52): C, 60.24; H, 4.63; N, 11.71; S, 6.70. Found: C, 59.88; H, 4.55; N, 11.65; S, 6.60.

*Methyl 5-chloro-3–(4-hydroxy-3,5-dimethoxybenzoyl)-1H-indole-2-carboxylate (****4****).* Was synthesised as **2** starting from **12** and 4-hydroxy-3,5-dimethoxybenzoyl chloride. Yield 11%, mp 118–120 °C (from ethanol). ^1^H NMR (CDCl_3_): *δ* 3.70 (*s*, 3H), 3.86 (*s*, 6H), 6.01 (br s, disappeared on treatment with D_2_O, 1H), 7.17 (*s*, 2H), 7.32–7.35 (*m*, 1H), 7.41–7.43 (*m*, 1H), 7.60–7.61 (*m*, 1H), 9.31 ppm (br s, disappeared on treatment with D_2_O, 1H). FT-IR (ATR): ν 1715, 3312 cm^−1^. Anal. calcd. for C_19_H_16_ClNO_6_ (389.79): C, 58.55; H, 4.14; N, 3.59; Cl, 9.09. Found: C, 58.36; H, 4.09; N, 3.23; Cl, 8.81.

*Methyl 5-chloro-3–(3,5-dimethoxybenzoyl)-1H-indole-2-carboxylate (****13****).* Was synthesised as **2** starting from **12** and 3,5-dimethoxybenzoyl chloride. Yield 21%, mp 185–188 °C (from ethanol). ^1^H NMR (CDCl_3_): *δ* 3.70 (*s*, 3H), 3.82 (*s*, 6H), 6.07 (*t*, *J* = 2.3 Hz, 1H), 7.0 (d, *J* = 4.7 Hz, 2H), 7.33–7.36 (*m*, 1H), 7.41–7.44 (*m*, 1H), 7.65–7.66 (*m*, 1H), 9.31 ppm (br s, disappeared on treatment with D_2_O, 1H). FT-IR (ATR): ν 1698, 3287 cm^−1^.

*Methyl 5-chloro-3–(3,5-dimethoxybenzyl)-1H-indole-2-carboxylate (****5****).* A mixture of **13** (0.1 g, 0.3 mmol), triethylsilane (0.072 g, 0.1 mL, 0.6 mmol) and trifluoroacetic acid (0.31 g, 0.21 mL, 3 mmol) was stirred at 25 °C for 48 h. The mixture was diluted with a saturated aqueous solution of NaHCO_3_ and extracted with ethyl acetate. The organic layer was washed with brine, dried and filtered. Removal of the solvent gave a residue that was purified by column chromatography (silica gel, *n*-hexane/ethyl acetate = 3:1 as eluent) to furnish **5** (0.05 g, 50%), mp 182–185 °C (from ethanol). ^1^H NMR (CDCl_3_): *δ* 3.71 (*s*, 6H), 3.92 (*s*, 3H), 4.43 (*s*, 2H), 6.30–6.31 (*m*, 1H), 6.41–6.44 (*m*, 2H), 7.25–7.35 (*m*, 2H), 7.58–7.60 (*m*, 1H), 8.80 ppm (br s, disappeared on treatment with D_2_O, 1H). FT-IR (ATR): ν 1690, 3323 cm^−1^. Anal. calcd. for C_19_H_18_ClNO_4_ (359.81): C, 63.43; H, 5.04; Cl, 9.85; N, 3.89. Found: C, 63.27; H, 4.97; Cl, 9.59; N, 3.70.

### Molecular modelling studies

**Figure 1.**
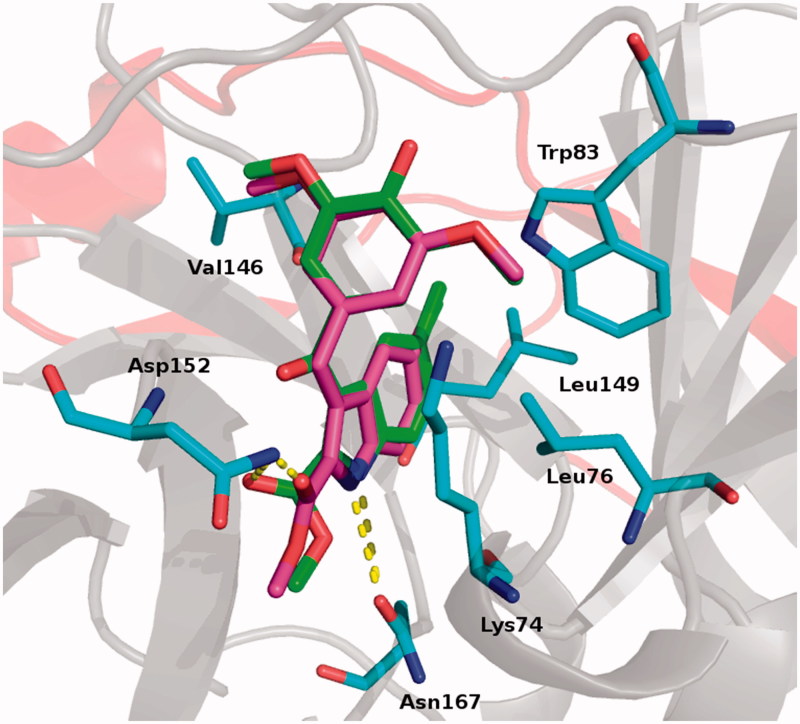
Proposed binding for derivatives **4** (green) and **5** (magenta). Residues involved in interactions are reported as cyan stick. H-bonds are shown as yellow dot lines. Protease is reported as cartoon: grey for NS3 and red for NS2 subunits.

All molecular modelling studies were performed on a MacPro dual 2.66 GHz Xeon running Ubuntu 12. The DENV protease structure (PDB ID: 2FOM)^27^ was downloaded from the protein data bank. An in-house compound library matching Lipinski’s rule of five^28^ was used as a training set. No pre-filter was applied. The docking simulations were performed with PLANTS^29^ using a 13 Å radius grid sphere. The centre of the binding site was settled according to Tamiri^30^. Molecules were scored by ChemPLP scoring function.^29^Figure 1 was generated by PyMOL^31^.

### Ethics statement and experimental animals

Six-day-old ICR strain-suckling mice were obtained from BioLasco Taiwan Co. Ltd (Taipei, Taiwan). All animal studies were conducted in specific pathogen-free conditions and carried out in accordance with the Guide for the Care and Use of Laboratory Animals. The experimental protocol was approved by the Animal Research Committee of Kaohsiung Medical University of Taiwan (IACUC, protocol number 102177) under the guidance of the Public Health Service (PHS) Policy on Humane Care and Use of Laboratory Animals.

### Cells and virus

Human hepatoma Huh-7 cells were cultured in Dulbecco’s modified Eagle’s medium (DMEM) containing 10% foetal bovine serum, 1% non-essential amino acids, and 1% antibiotic–antimycotic within 5% CO_2_ supplement at 37 °C. *Aedes albopictus* C6/36 mosquito cells were cultured in RPMI1640 medium containing 10% fetal bovine serum, 1% non-essential amino acids, 1% antibiotic–antimycotic and 1% sodium pyruvate within 5% CO_2_ supplement at 37 °C. DENV-2 (DENV type-2 strain 16681) was amplified in C6/36 mosquito cells. *Spodoptera frugiperda* (Sf9) insect cells were cultured in Grace’s medium with 10% hear-inactivated foetal bovine serum (FBS) and 1% antibiotic–antimycotic at 26 °C.

### Evaluation of anti-DENV RNA activity

Huh-7 cells were seeded in 24-well plate and infected DENV at an MOI of 0.2 for 2 h and followed by test compound treatment at concentration of 0, 1, 5, 10, 25, 50 and 100 µM for 3 days. The total cellular RNA was harvested by RNA extraction kit^32^ following manufacturer’s instrument. DENV RNA and cellular mRNA levels were determined by quantitative real-time reverse-transcription polymerase chain reaction (RT-qPCR) with specific primers^33^. The DENV RNA level was normalised by cellular glyceraldehydes-3-phosphate dehydrogenase (GAPDH) mRNA level. The relative DENV RNA level was calculated by StepOne™ Software v2.2.2 (Applied Biosystems, Foster City, CA, USA) following normalisation of cellular glyceraldehydes-3-phosphate dehydrogenase (GAPDH) mRNA level. The zero dose was defined as 100%. The EC_50_ values were calculated from non-linear regression curve fitting using GraphPad Prism7 software (San Diego, CA, USA). Results were obtained from three independent experiments.

### Cell cytotoxicity assay

The Huh-7 cells were seeded in 96-well and treated with test compound at concentration of 0, 10, 25, 50, 100, 150 and 200 µM. After 3 days incubation, the cell viability was determined by CellTiter 96 AQueous One Solution Cell Proliferation Assay (MTS assay) as previously reported^34^. Briefly, the MTS assay buffer was added to the cultured plate following removal of the culture medium. After 2 h of incubation at 37 °C, the absorbance at 492 nm was measured. The cell viability was calculated from non-linear regression curve fitting using GraphPad Prism7. The zero dose was defined as 100%. Results were obtained from three independent experiments.

### Purification of DENV NS5 protein

The DENV-2 NS5 protein purification was performed as previously described^33^. Briefly, Sf9 cells were infected by the recombinant baculovirus vAc-DENV-NS5 at an MOI of 10 for 3 days. The cells were pelleted by centrifugation at 1000×*g* for 5 min at 4 °C and washed twice with phosphate-buffered saline (PBS). The cells were suspended in 5 mL of binding buffer (20 mM sodium phosphate, pH 7.5, 300 mM NaCl, 20 mM imidazole) containing protease inhibitor cocktail, and then disrupted by sonication. After centrifugation, the supernatant containing His-tagged DENV-2 NS5 protein was subjected to metal affinity chromatography employing the AKTA prime protein purification system^35^. The eluted DENV-2 NS5 protein was concentrated by an Amicon Ultra-15 30 k centrifugal filter device^36^ and then dialysed against PBS overnight at 4 °C.

### Evaluation of anti-DENV RdRp activity

The cell-based experiments were performed as previously described^33^. Briefly, the Huh-7 cells were transfected with 0.5 µg of p(+)RLuc-(–)DV-UTRΔC-FLuc and DENV NS5 expression vector pcDNA-NS5-Myc followed by compound treatment for 3 days. The RLuc and FLuc activities were analysed by Dual-Glo Luciferase Assay System^37^. The enzyme-based fluorescence-based alkaline phosphatase-coupled polymerase assay (FAPA) was performed as previously described^33^. Briefly, the template was amplified from the cDNA of DENV-2 minus strand 3′-UTR and its RNA was synthesised by the T7 Megascript kit^38^. The 100 nM DENV NS5 protein was incubated with 1, 5 or 10 µM test compound, 100 nM (−) 3′-UTR RNA, 20 µM CTP, GTP, UTP and 20 µM BBT-ATP (Jena Bioscience) in a total volume of 30 µL in assay buffer (50 mM Tris-HCl, pH 7.5, 10 mM KCl, 1 mM MgCl_2_, 0.3 mM MnCl_2_, 0.001% Triton X-100 and 10 µM cysteine) at 25 °C for 60 min. The 30 µL of 2.5× stop buffer (200 mM NaCl, 25 mM MgCl_2_, 1.5 M diethanolamine, pH 10) with 25 nM calf intestinal alkaline phosphatase (Promega) was added to the reaction after 60-min incubation. The fluorescence signal was measured at excitation wavelength of 422 nm and emission wavelength of 566 nm, respectively.

### Purification of DENV NS2B/NS3pro protein

The DENV NS2B/NS3pro purification was performed as previously described^39^. Briefly, the pET24b-NS2B-NS3pro plasmid was transformed into competent *Escherichia coli* BL21 (DE3) and grew in kanamycin (50 µg/mL)-containing LB at 26 °C until OD600 nm reached 0.6. The isopropyl-β-D-thiogalactopyranose was added into the bacterial culture at 0.5 mM for protein induction. After 12-h incubation, cells were collected and subjected to protein purification following previous description.

### Evaluation of anti-DENV protease activity

For cell-based activity assay, the Huh-7 cells were cotransfected with 0.5 µg of pEG(MITA)SEAP reporter vector and DENV protease expression vector pcDNA-NS2B-GSG-NS3-Myc followed by compound treatment for 3 days. The SEAP activity was analysed by Phospha-Light SEAP Reporter Gene Assay System^40^. The enzyme-based experiment was performed as previously described^39^. Briefly, the 7-amino-4-methylcoumarin (AMC) fluorophore-linked peptide substrate Boc-GRR-AMC^41^ and purified DENV NS2B/NS3pro protein were used to analyse DENV NS2B/NS3 protease activity. The 100 nM DENV NS2B/NS3pro protein was incubated with 1, 5 or 10 µM tested compound and 10 µM Boc-GRR-AMC in cleavage buffer (200 mM Tris [pH 9.5], 20% glycerol) for 30 min at 25 °C. The fluorescence signal was detected at excitation wavelength of 380 nm and emission wavelength of 465 nm, respectively.

### Evaluation of anti-DENV activity by ICR-suckling mice

Breeder ICR mice were purchased from BioLasco Taiwan Co. Ltd. The experimental protocol was approved by the Animal Research Committee of Kaohsiung Medical University of Taiwan (IACUC, protocol number 102177). All mice received humane care and fed with standard rodent chew and water *ad libitum*. Mice were kept under a standard laboratory condition following the Animal Use Protocol of Kaohsiung Medical University. The 6-day-old ICR-suckling mice were intracerebrally injected with DENV-2 followed by intracerebrally injecting saline or test compound at 1, 3, 5 days postinfection (dpi). Mice intracerebrally injected with 65 °C heat-inactive DENV-2 followed with saline treatment served as healthy control. The body weight, clinical scores and survival rate were recorded every day. The mice were sacrificed at 6 dpi.

## Results and discussion

### Chemistry

**Scheme 1.**
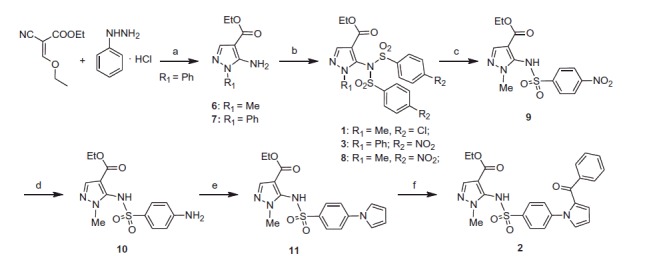
Synthesis of the final compounds **1–3**. (a) TEA, reflux, 7 h. (b) 4-Cl- or 4-NO_2_-C_6_H_4_SO_2_Cl, pyridine, 60 °C, overnight. (c) LiOH, 50 °C, 15 min. (d) SnCl_2_, 80 °C, 3 h. (e) 2,5-(OMe)_2_-THF, 100 °C, 1 h. (f) benzoyl chloride, AlCl_3,_ closed vessel, 110 °C, 70 W, 4 min.

Compounds **1–3** were synthesised as shown in Scheme 1. Ethyl 5-amino-1-methyl-1*H*-pyrazole-4-carboxylate (**6**) was purchased from Maybridge. Treatment of ethyl 2-cyano-3-ethoxyacrylate with phenylhydrazine hydrochloride yielded ethyl 5-amino-1-phenylpyrazole-4-carboxylate (**7**)^26^. Pyrazoles **6** and **7** were converted into final derivatives **1**, **3** or intermediate **8** by reaction with 4-chloro- or 4-nitrobenzenesulphonyl chloride, respectively, in pyridine. Treatment of **8** with lithium hydroxide in aqueous tetrahydrofuran for 15 min gave the sulphonamide **9**. Tin (II) chloride dihydrate reduction of **9** for 3 h in boiling ethyl acetate afforded amino derivative **10** that was converted into pyrrole derivative **11** with 2,5-dimethoxytetrahydrofuran in glacial acetic acid at 80 °C for 1 h. Derivative **2** was prepared from **11** by MW-assisted reaction with benzoyl chloride in the presence of anhydrous aluminium chloride in 1,2-dichloroethane at 110 °C (70 W) for 3 min.

**Scheme 2.**
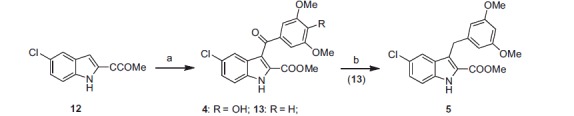
Synthesis of the final compounds **4** and **5**. (a) 4-OH-3,5-OMe_2_- or 3,5-OMe_2_-C_6_H_4_COCl, AlCl_3_, closed vessel, 110 °C, 70 W, 2 min. (b) TES, TFA, 48 h, rt.

Indole derivatives **4** and **5** were synthesised as shown in Scheme 2. Final compound **4** and the intermediate **13** were prepared by MW-assisted aroylation of methyl 5-chloro-1*H*-indole-2-carboxylate (**12**) with 4-hydroxy-3,5-dimethoxybenzoyl chloride or 3,5-dimethoxybenzoyl chloride, respectively, in the presence of anhydrous aluminium chloride in 1,2-dichloroethane at 110 °C (70 W) for 2 min. Treatment of **13** with triethylsilane in trifluoroacetic acid for 48 h afforded the corresponding methylene compound **5**.

### Virtual screening studies

In search for allosteric inhibitors of the NS2B/NS3 protease, we carried out virtual screening (VS) and docking studies. We focused our VS studies against the allosteric site^,^ of the enzyme^42^^,^^43^. To our knowledge, only few protease allosteric inhibitors have been reported^30^, and many of them are natural compounds that do not possess drug-like properties^30^. We performed structure-based screening studies on an open conformation of the NS2B/NS3 protease structure^44^ (pdb code: 2FOM)^27^ available at the protein data bank webserver^45^. This structure of the NS2B/NS3 protease was previously used in VS campaign in search for new allosteric inhibitors^43^.

Compounds **4** and **5** shared virtually superimposable binding modes: (a) the 2-methoxycarbonyl ester occupied the inner part of the binding pocket and was stabilised by an H-bond with Asp152 side chain; (b) the indole NH formed an H-bond with Asn167; (c) the indole ring arranged a series of hydrophobic contacts with Val146, Leu149 and Leu76; (d) the methoxylphenyl moiety of both inhibitors formed hydrophobic interactions with Trp83 and a pi-cation interaction with Lys74. (Figure 1).

### Biological activity

#### DENV NS5 RdRp inhibitors

**Figure 2.**
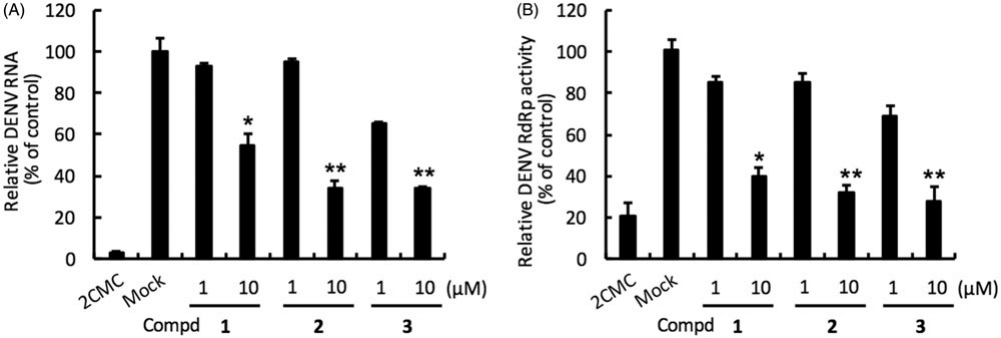
Inhibition of DENV RNA replication and RdRp activity by compounds **1–3**. Panel A. Huh-7 cells were infected with DENV-2 at a multiplicity of infection (M.O.I) of 0.2 and followed by treatment with the DENV polymerase inhibitor for 3 days. The DENV RNA level was analysed by RT-qPCR with specific primer targeting viral NS5 gene, and relative viral RNA levels were normalised against cellular GADPH mRNA levels. Treatment of 50 μM 2′-C-methylcytidine (2CMC) direct against DENV RdRp served as positive control. 0.1% DMSO (Mock) served as negative control. Panel B. Huh-7 cells were transfected with pEG(MITA)SEAP and pcDNA-NS2B-GSG-NS3-Myc followed by incubation with each compound. The luciferase activity was analysed after 3 days treatment. Error bars denote the means ± SD of three independent experiments. *p < .05; **p < .01.

Compounds **1–3** were evaluated in DENV-2 infected Huh-7 cells by determining DENV RNA levels using specific qRT-PCR primer targeting viral NS5 levels, normalised to cellular GADPH mRNA levels. Compound **1** inhibited the DENV-2 with EC_50_ of 11.09 ± 0.21 µM and selectivity index (SI = CC_50_/EC_50_ ratio) of 17.7. Compound **2** bearing a benzoyl tail at position 2 of the pyrrole potently inhibited DENV-2 with EC_50_ of 7.61 ± 0.36 µM and SI >26.3. 1-Phenylpyrazole derivative compound **3** with the 4-nitrophenyl group showed potent anti-DENV-2 activity with EC_50_ of 5.66 ± 0.25 µM and SI > 35.3. Collectively, compounds **1–3** strongly reduced DENV RNA level (Table 1 and Figure 2, panel A).

To confirm the anti-RdRp activity of **1–3**, we performed a cell-based RdRp reporter assay. The Huh-7 cells were transiently expressed p(+)RLuc-(−)DV-UTRΔC-FLuc and DENV NS5 expression vector pcDNA-NS5-Myc and then treated with the tested compound. Our data showed that compounds **1–3** efficiently inhibited DENV NS5 RdRp activity with EC_50_ of 8.07 ± 0.26, 7.22 ± 0.38 and 5.99 ± 0.30 µM (Table 1 and Figure 2, panel B). To further confirm the inhibitory specificity of compounds **1–3** against NS5 RdRp activity, we performed an enzyme-based FAPA assay. Our data showed that compounds **1–3** exhibited direct-action against DENV RdRp activity (Table 1) with EC_50_ of 7.78 ± 0.31, 5.34 ± 0.17 and 4.87 ± 0.24 µM, respectively, whose values correlated with results of cell-based assay. Taken together, these results demonstrated that compounds **1–3** attenuate DENV replication by directly inhibiting DENV RdRp activity.

#### DENV NS3 protease inhibitors

**Figure. 3.**
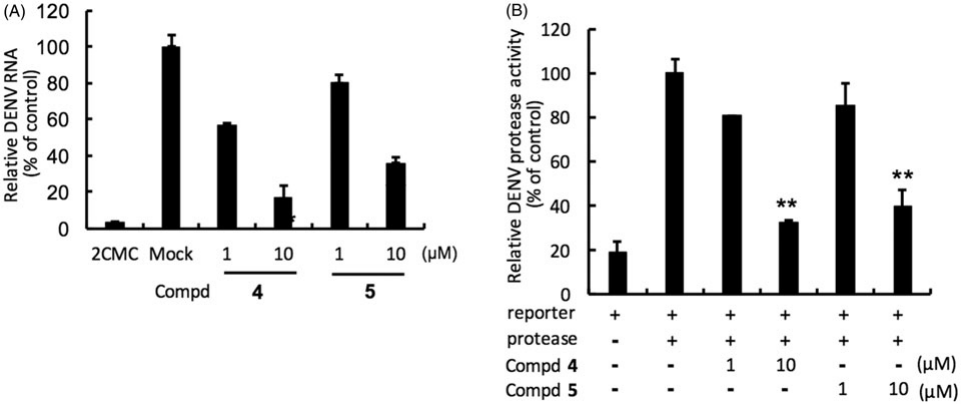
Inhibition of DENV RNA and NS3-protease activity by compounds **4** and **5**. Panel A. Huh-7 cells were infected with DENV-2 at a multiplicity of infection (M.O.I) of 0.2 and followed by the treatment of each DENV protease inhibitors for 3 days. The DENV RNA level was analysed by RT-qPCR with specific primer targeting viral NS5 gene, and relative viral RNA levels were normalised against cellular GADPH mRNA levels. Treatment of 50 μM 2′-C-methylcytidine (2CMC) direct against DENV RdRp served as positive control. 0.1% DMSO (Mock) served as negative control. Panel B. Huh-7 cells were transfected with pEG(MITA)SEAP and pcDNA-NS2B-GSG-NS3-Myc followed by incubation of each compounds, and the luciferase activity was analysed after 3 days treatment. Error bars denote the means ± SD of three independent experiments. *p < .05; **p < .01.

Huh-7 cells were infected with DENV-2 and then treated with protease inhibitors for 3 days and DENV RNA synthesis was evaluated with specific qRT-PCR primers targeting viral NS5 gene. Compounds **4** and **5** strongly inhibited the DENV-2 DNA replication with EC_50_s of 4.60 ± 0.03 µM and 7.28 ± 0.13 µM, respectively (Table 2 and Figure 3, panel A). In addition, a cell-based DENV protease reporter assay was used to characterise the protease specificity of compounds **4** and **5**. Huh-7 cells were transfected with pEG(MITA)SEAP and DENV protease expression vector pcDNA-NS2B-GSG-NS3-Myc and then treated with **4** or **5**. Both compounds showed strong inhibitory activity against the DENV protease with EC_50_ of 6.71 ± 0.20 µM and 7.92 ± 0.62 µM, respectively (Table 2 and Figure 3, panel B). We further performed an enzyme-based assay to evaluate the inhibitory specificity of compounds **4–5**. The data showed that compounds **4–5** specifically suppressed DENV protease activity with EC_50_ of 4.72 ± 0.3 µM and 6.90 ± 0.11 µM, respectively (Table 2). In conclusion, the results indicated that both compound **4** and **5** suppress DENV replication by directly inhibiting DENV protease activity.

#### Evaluation of 3 and 4 in DENV-infected ICR-suckling mouse model

**Figure 4.**
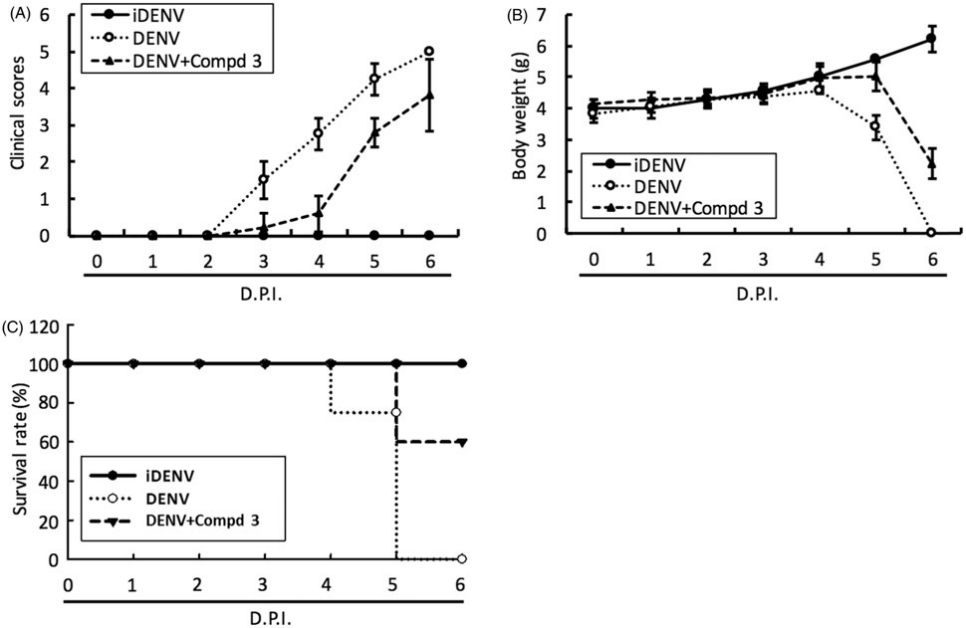
DENV RdRp inhibitor **3** protected ICR-suckling mice from DENV infection. 6-day-old ICR-suckling mice were intracerebrally injected with heat-inactive DENV (iDENV, *n* = 5) or active DENV (DENV, *n* = 4). Mice-receiving DENV were treated with 10 mg/kg of compound **3** (*n* = 5) at 1, 3, 5 dpi. Panel A, clinical scores; Panel B, body weight and Panel C, survival rate were recorded every day. Disease severity was scored as follow: 0: healthy, 1: slightly sick (reduced mobility), 2: inappetance, 3: weight loss and difficult to move, 4: paralysis, 5: death. Each group included 6 mice. Error bars denote the means ± SD.

**Figure 5.**
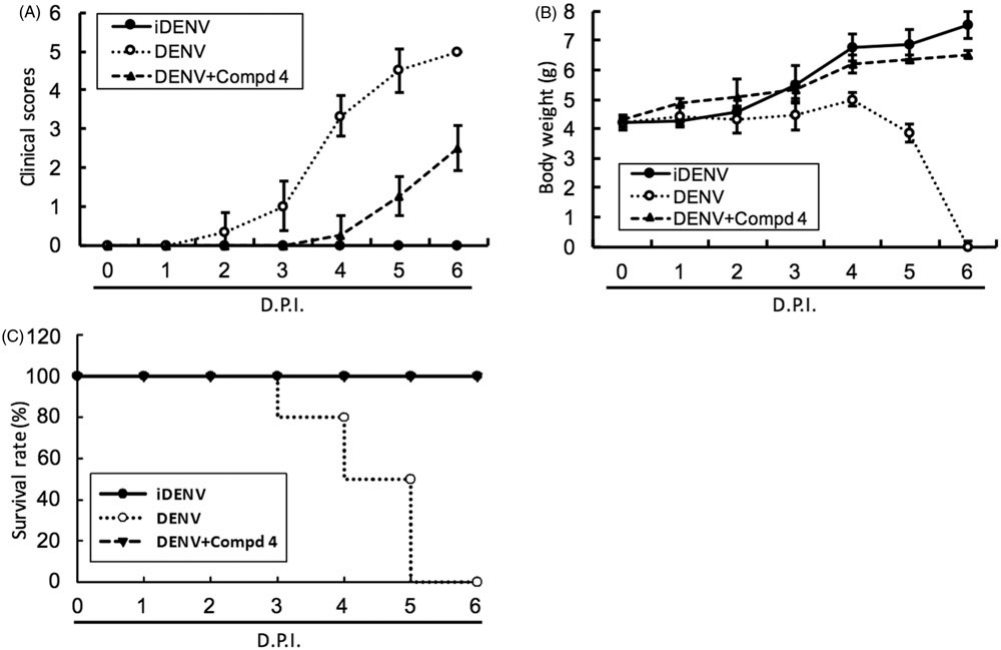
DENV protease inhibitor **4** protected ICR-suckling mice from DENV infection. Six-day-old ICR-suckling mice were intracerebrally injected with heat-inactive DENV (iDENV, *n* = 6) or active DENV (DENV, *n* = 6). Mice-receiving DENV were treated with 1 mg/kg of compound **4** (*n* = 6) at 1, 3, 5 dpi. Panel A, clinical scores, Panel B, body weight, and Panel C, survival rate were recorded every day. Disease severity was scored as follow: 0: healthy, 1: slightly sick (reduced mobility), 2: inappetance, 3: weight loss and difficult to move, 4: paralysis, 5: death. Each group included six mice. Error bars denote the means ± SD.

To access the antiviral activity of compound **3** and **4***in vivo*, 6-day-old ICR-suckling mice were inoculated with 2.5 × 10^5^ pfu of DENV-2 by intracerebral injection and then were administered with compound **3** (10 mg/kg) or **4** (1 mg/kg) by intracerebral injection at 1-, 3- and 5-day postinfection (dpi); inoculation of heat-inactivated DENV-2 (iDENV) served as a mock control. The results of clinical scores (Figures 4(A) and 5(A)) and body weight (Figures 4(B) and 5(B)) showed that compound **3** or compound **4** treatment reduced DENV-induced pathology, including ruffled fur, anorexia, severe paralysis, lethargy, on DENV-infected mice within 4 to 6 dpi. Furthermore, compound **3** or **4** treatment significantly protected the mice against life-threatening DENV infection, as compared to the untreated mice (Figures 4(C) and 5(C)). Collectively, these results demonstrated that both classes of small molecules, targeting polymerase and protease activities, inhibited DENV replication and represent prototypical DAA molecules for further development through compound modification.

#### Synergistic effect of NS5 RdRp inhibitor 3 and NS3 protease inhibitor 4 against DENV replication

**Figure 6.**
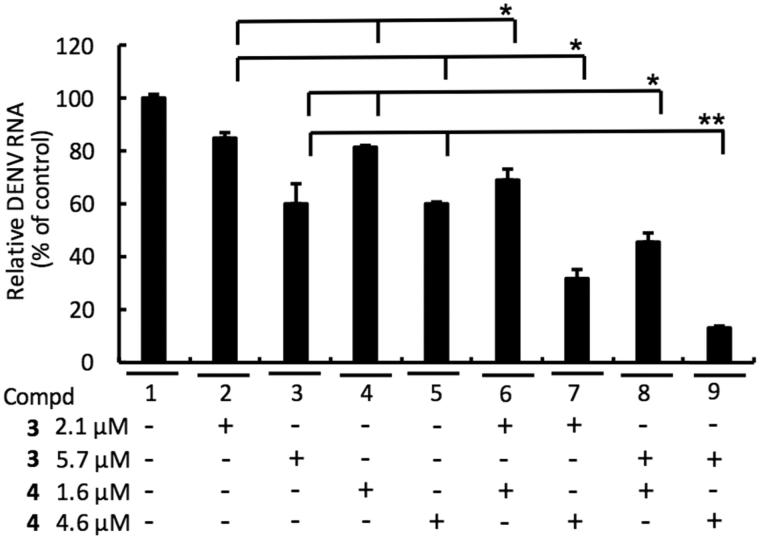
Combination assay of RdRp (**3**) and NS3 protease (**4**) inhibitors. Huh-7 cells were infected with DENV-2 at a multiplicity of infection (M.O.I) of 0.2 and followed by treatment of **3** and **4** with indicated concentration for 3 days. The DENV RNA level was analysed by RT-qPCR with specific primer targeting viral NS5 gene, and relative viral RNA levels were normalised against cellular GADPH mRNA levels. Error bars denote the means ± SD of three independent experiments. *p < .05; **p < .01.

To examine whether combinational treatment of NS5 RdRp inhibitor **3** and NS3 protease inhibitor **4** could synergistically inhibit DENV replication, DENV-2-infected Huh-7 cells were cotreated with **3** and **4** at the indicated concentration for 3 days. As shown in Figure 6, a synergistic inhibition of DENV replication was observed with the **3** and **4** combination (lines 6–9), as compared to single-compound treatment (lines 2–5).

## Conclusions

We sought to design and characterise potential anti-DENV inhibitors by targeting the viral enzymatic, NS5 RNA-dependent RNA polymerase and the NS3 protease activities. We identified five potential compounds demonstrating anti-DENV replication activity without cytotoxicity. Two compounds targeting DENV NS3 protease and NS5 RdRp also exhibited anti-DENV activity in ICR-suckling mouse model of DENV infection. Combination treatment exhibited enhanced inhibition of DENV replication. Taken together, these results demonstrate that both classes of small molecules inhibited DENV replication and represent prototypical DAA molecules for further development through compound modification.
